# The running economy and biomechanics of competitive distance runners wearing advanced footwear technology spikes

**DOI:** 10.1007/s00421-026-06257-y

**Published:** 2026-05-26

**Authors:** Bradley J. Needles, Sewan Kim, Alena M. Grabowski

**Affiliations:** 1 Applied Biomechanics Laboratory, Integrative Physiology, University of Colorado Boulder, Boulder, CO USA; 2 Applied Exercise Science Laboratory, Integrative Physiology, University of Colorado Boulder, Boulder, CO USA

**Keywords:** Metabolic power, Track and field, Athletics, Distance running

## Abstract

**Purpose:**

The aim of this study was to investigate the running economy and biomechanics of runners wearing Advanced Footwear Technology (AFT) spiked shoes (spikes) and AFT shoes relative to traditional spikes.

**Methods:**

17 (7 female & 10 male) competitive distance runners completed two sessions of eight (four shoes × two replicates), five-minute trials at 15 km*hr^−1^ (females) or 17 km*hr^−1^ (males) wearing Nike ZoomX Dragonfly (NDF) and On Cloudspike 10,000 m (OCS) AFT spikes, Nike Vaporfly Next % 2 (NVN) AFT shoes, and Nike Zoom Victory 3 (NZV) traditional spikes. We measured metabolic rates and ground reaction forces and compared average running economy (W*kg^−1^) and biomechanical variables from four trials per shoe per runner.

**Results:**

When runners wore NDF and OCS AFT spikes and NVN AFT shoes, running economy improved by 2.1%, 2.3%, and 1.9%, respectively, compared to NZV traditional spikes (p < 0.001). These improvements coincided with 6.1%, 4.5%, and 8.9% increases in leg stiffness and 1.5%, 2.2%, and 2.3% increases in ground contact time for the NDF, OCS, and NVN, respectively, compared to NZV traditional spikes (p < 0.002).

**Conclusion:**

Running economy improved by 2.1% for athletes wearing compliant AFT spikes and shoes compared to traditional spikes. Future generations of AFT spikes that are designed with further increases in midsole compliance and energy return could improve race performance by eliciting potentially favorable biomechanical changes, such as increased leg stiffness and/or ground contact time.

## Introduction

Since the introduction of Advanced Footwear Technology (AFT) spiked racing footwear for track and field (spikes) in 2020, elite runners have improved their race times by 0.3 to 2.6% for the 800 m to 10,000 m events compared to race times achieved for the same events while wearing traditional spikes (Willwacher et al. 2024; Needles and Grabowski 2024). These improvements in race times are likely linked to the properties of the midsole foams used to create AFT spikes. AFT spikes are characterized by more compliant (less stiff) and resilient (greater energy return) midsole foams, increased midsole stack height (thickness), and the inclusion of a midsole embedded rigid plate compared to traditional spikes (Healey et al. 2022; Frederick 2022). AFT midsole foams, such as polyether block amide (PEBA), are two times more compliant and return 120% more mechanical energy per step than ethylene–vinyl acetate (EVA) midsole foams used in traditional spikes (Hoogkamer et al. 2018). Thus, race performance improvements for athletes wearing AFT spikes may be due to physiological and biomechanical changes elicited by the increased compliance and energy return of AFT spikes compared to traditional spikes.

A key factor in distance-running race performance is running economy, a measure of the energy produced by an athlete’s aerobic metabolism while running at a given speed (Kipp et al. 2019; Joyner 1991; Coyle 1995; Barnes and Kilding 2015). Traditionally, running economy has been expressed in units of milliliters of oxygen per kilogram of body mass per minute [ml O_2_*kg^−1^*min^−1^] (Daniels and Daniels 1992). However, to account for changes in metabolic substrate utilization, running economy has also been defined as body mass normalized gross metabolic power at a given speed [W*kg^−1^] (Kipp et al. 2019; Hoogkamer et al. 2018, 2016; Fletcher et al. 2009). Previous studies have compared the running economy of athletes wearing AFT footwear and traditional racing footwear to gain insight into the potential performance benefits of wearing AFT spikes and shoes (Barnes and Kilding 2019; Hoogkamer et al. 2018; Hunter et al. 2019; Joubert et al. 2024). It has been reported that runners wearing AFT shoes designed for road racing improved their running economy by 2 to 4% compared to traditional racing footwear when running at 14 to 18 km*hr^−1^ (Barnes and Kilding 2019; Hoogkamer et al. 2018; Hunter et al. 2019). Since AFT spikes are constructed with the same midsole foams as AFT shoes, it has been hypothesized that AFT spikes may improve running economy as well. Indeed, Joubert et al. found a 2.1% improvement in running economy for participants running at 16 km*hr^−1^ while wearing AFT spikes compared to traditional spikes (Joubert et al. 2024). These findings indicate that athletes wearing AFT spikes may achieve improved running economy, leading to improved race performance compared to traditional spikes.

Despite studies showing the running economy and race performance benefits of wearing AFT footwear (Willwacher et al. 2024; Needles and Grabowski 2024; Hoogkamer et al. 2018; Rodrigo-Carranza et al. 2025; Barnes and Kilding 2019; Hunter et al. 2019; Bermon et al. 2021), the underlying mechanism or mechanisms that drive these benefits are debated (Healey et al. 2022; Hoogkamer et al. 2018, 2019). One possible source of improved running economy for athletes wearing AFT spikes is increased leg stiffness, defined as the peak ground reaction force applied by the leg divided by the peak axial compression of the leg (Farley et al. 1993; Blickhan 1989; McMahon and Cheng 1990; Cavagna et al. 1964). Previous studies have reported improvements in race performance and running economy along with increased leg stiffness while athletes ran on compliant surfaces. Kerdok et al. found that a decrease in treadmill surface stiffness from 945.7 N*mm^−1^ to 75.4 N*mm^−1^ resulted in a 29% increase in leg stiffness and a 12% improvement in running economy when participants ran at 13.3 km*hr^−1^ (Kerdok et al. 2002). Moreover, while racing on a “tuned” track surface that was 4.5 times more compliant than traditionally constructed indoor tracks (195 N*mm^−1^ versus 875 N*mm^−1^), runners improved their 440 yard to 2 mile race times by 2.9% compared to racing at other venues (McMahon and Greene 1978, 1979). These findings suggest that increased leg stiffness may lead to improved running economy and, as a result, improve race performance for runners wearing compliant AFT spikes and shoes compared to traditional spikes. However, the relationship between leg stiffness and running economy while wearing AFT spikes has not yet been established.

Similar to leg stiffness, increased ground contact time during the stance phase of running may improve running economy for athletes wearing AFT spikes and shoes. After observing a consistent relationship between running economy and ground contact time for terrestrial animals, Kram and Taylor proposed the “Cost of Generating Force Hypothesis” where the body mass normalized metabolic power of running [W*kg^−1^] is inversely related to ground contact time (Kram and Taylor 1990). In a study of prototype AFT shoes, Hoogkamer et al. reported increased ground contact time and improved running economy for participants wearing AFT shoes compared to traditional road racing shoes while running at 14, 16, and 18 km*hr^−1^ (Hoogkamer et al. 2018). Moreover, while running at 14 and 16 km*hr^−1^, ground contact times were 1.6 to 2.5% longer and running economy improved by 2.4 to 3.1% for runners wearing AFT shoes compared to traditional spikes (Barnes and Kilding 2019). However, follow up studies conducted by Hunter et al. and Hoogkamer et al. found no significant difference in ground contact time between athletes using AFT and traditional shoes (Hunter et al. 2019; Hoogkamer et al. 2019). Thus, despite the hypothesized relationship between running economy and ground contact time, previously published research has reported mixed results for ground contact time while runners wear AFT shoes compared to traditional racing footwear.

The goal of this study was to quantify the physiological and biomechanical effects of competitive distance runners wearing AFT spikes and shoes compared to traditional spikes. By understanding the material characteristics of AFT footwear and the physiological and biomechanical changes that occur while athletes run in AFT spikes and shoes compared to traditional spikes, we aimed to provide insights for the development of future iterations of AFT spikes that could further improve race performance. In the present study, we utilized a force measuring treadmill with a running surface capable of accommodating spikes to measure metabolic rates and ground reaction forces of competitive distance runners wearing AFT spikes, AFT shoes, and traditional spikes. Additionally, we quantified the midsole properties of AFT spikes, AFT shoes, and traditional spikes to explore the potential relationships between footwear midsole characteristics, running economy and biomechanics.

Since numerous studies have shown that running economy improves while running on compliant surfaces or wearing compliant footwear (Rodrigo-Carranza et al. 2025; Hoogkamer et al. 2018; Hunter et al. 2019; Barnes and Kilding 2019; Kerdok et al. 2002), we hypothesized that running economy would improve when participants wore AFT spikes and shoes compared to traditional spikes. Based on previous studies of AFT shoes, increased leg stiffness and ground contact time may partially explain the improved running economy when runners wear AFT spikes and shoes (Biewener et al. 2004; Kram and Taylor 1990). Therefore, we hypothesized that leg stiffness and ground contact time would increase when participants wore AFT spikes and shoes compared to traditional spikes.

## Methods

### Participants

17 (7 female and 10 male) competitive distance runners participated (Table 1). We defined competitive distance runners as runners who had run a 10-km race at sea level faster than 37 min for females and 32 min for males or achieved an equivalent performance in a different distance running event within the past two years. Competitive distance runners were selected as our participant population since they represent the target user of AFT spikes and shoes. We conducted an a priori sample size calculation (effect size: 0.45, significance level: 0.05, power: 0.80) using RStudio (Boston, MA, USA) and found that a sample of 15 participants would result in a statistical power of 0.80 with an α of 0.05 to detect a significant difference in running economy, leg stiffness, and ground contact time. To account for potential participant drop outs, we recruited 17 participants but experienced no drop outs. The participants all wore one of the following shoe sizes: US Women’s 7, 8, or 9 or US Men’s 9, 10, or 11. Prior to participating in the study, each participant provided informed written consent. The study was performed in accordance with the ethical standards of the Declaration of Helsinki. Ethics approval was obtained from the University of Colorado Institutional Review Board (Protocol# 23-0063).

**Table 1:** Anthropometric and physiological characteristics of 17 competitive distance runners

	Age [years]	Mass [kg]	Height [cm]	Leg length [cm]	$\dot{V}$O_2max_ [ml O_2_*kg^−1^*min^−1^]
All Athletes (n = 17)	25.5 ± 3.2	61.8 ± 7.8	173.5 ± 7.3	92.7 ± 3.7	61.6 ± 6.0
Female (n = 7)	26.9 ± 2.6	54.8 ± 5.4	166.6 ± 3.8	90.3 ± 2.8	56.9 ± 4.0
Male (n = 10)	24.5 ± 3.2	66.7 ± 4.9	178.3 ± 5.0	94.4 ± 3.2	65.0 ± 4.7

Values are reported as mean ± SD

### Footwear conditions

The footwear conditions included in the present study were selected to represent a selection of the competition footwear choices available to track athletes racing 800 m to 10,000 m distances in 2021. Additionally, we included an AFT shoe condition to allow for comparison between AFT spikes and AFT shoes and to relate our results to previously published AFT shoe research. Participants ran wearing two AFT spike conditions that included a rigid midsole plate with 20 mm and 25 mm of PEBA midsole foam (Nike ZoomX Dragonfly (NDF) and On Cloudspike 10,000 m (OCS), respectively), an AFT shoe that included a rigid midsole plate with 40 mm of PEBA midsole foam (Nike VaporFly Next % 2 (NVN)), and a traditional track spike that included 10 mm of EVA midsole foam (Nike Zoom Victory 3 (NZV)). The mass of the NDF, OCS, NVN, and NZV, when averaged across all shoe sizes included in the study, was 263, 300, 372, and 229 g per pair, respectively. We ensured that none of the shoes used in the study accumulated more than 70 km of wear during the study since the distance run in a shoe may alter its function (Rodrigo‐Carranza et al. 2024).

### Footwear midsole characteristics testing

Due to the potential role of midsole material characteristics in running economy improvements for athletes wearing AFT spikes and shoes (Hoogkamer et al. 2018; Healey et al. 2022; Burns and Joubert 2024), we used a materials testing machine (MTM) (Instron Series 5859, Norwood, MA) to measure the compression of each footwear condition’s midsole under a vertical compressive load. From the vertical force applied and resulting compression of the midsole, we estimated the vertical stiffness and mechanical energy return of each footwear condition based on the magnitude of force applied during the stance phase of running.

#### Vertical stiffness

**Fig. 1:**
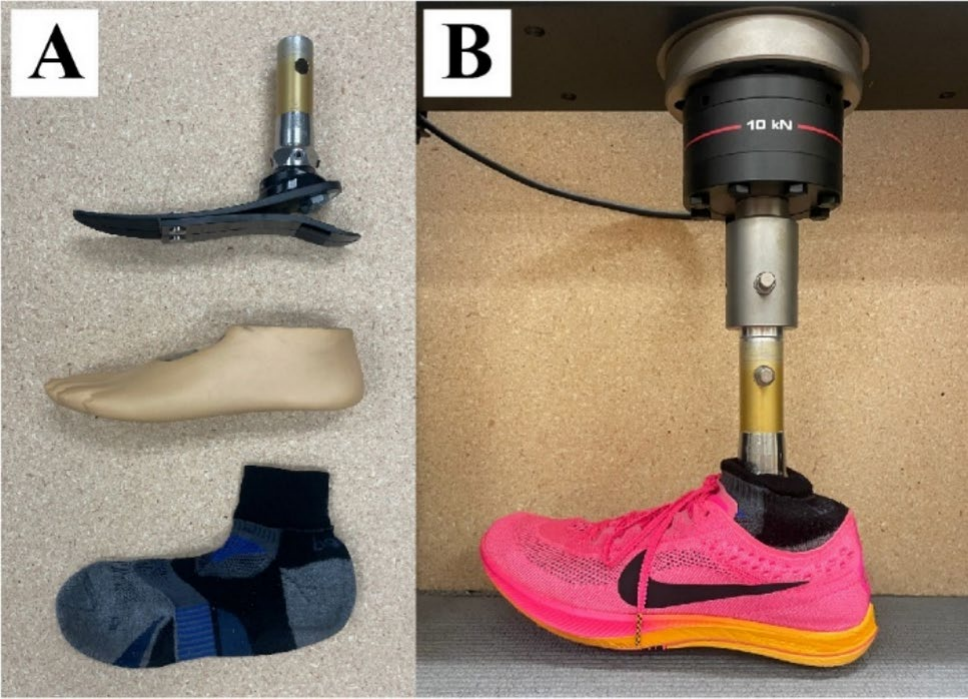
**A** We used an Össur low-profile Vari-flex prosthetic foot attached to a pylon, a cosmesis, and an athletic sock to measure the vertical stiffness of each shoe or spike. **B** We placed the prosthetic foot within each spike or shoe condition and attached the prosthetic foot to the materials testing machine. A 10 mm thick Mondo track surface was placed under the spike or shoe during testing to simulate the intended running surface

Stiffness measurements are proportional to the area of force application for point elastic materials such as footwear midsole foams (Nigg and Yeadon 1987), and previous studies have reported that, regardless of foot strike pattern, a runner’s foot is roughly parallel to the ground at the time of peak vertical ground reaction force production during level ground running (Ferris et al. 1998; Clark et al. 2016). Thus, to measure footwear stiffness we elected to emulate a biological foot using a prosthetic foot (Össur low-profile Vari-flex, Stiffness Category 5, Size 26) fitted with a cosmesis and an athletic sock (Fig. 1A) and applied a vertical force on a US men’s size 11 version of each footwear condition. Additionally, we aligned the sole of each footwear condition parallel to the testing platform and aligned a pylon, which was attached to the prosthetic foot, to the MTM perpendicular (within 0.5°) to the testing platform (Fig. 1B). To replicate the intended training and competition surface for AFT and traditional spikes, we placed a section of 10 mm thick Mondo track surface (Mondo Flooring, Alba, Italy) underneath the footwear during testing (Fig. 1B).

Previous research has reported peak vertical ground reaction force (vGRF_peak_) values ranging from 2.9 to 3.1 units of body weight (BW) for male participants running at 14 to 18 km*hr^−1^ while wearing prototype NVN AFT shoes (Hoogkamer et al. 2018). Since we recruited female and male participants with body mass ranging between 45 and 75 kg, we anticipated our participants would produce vGRF_peak_ values ranging between approximately 1300 and 2400 N. Accordingly, we began the vertical stiffness testing by preloading each footwear condition with 4 N and applied a compressive vertical force to the prosthetic foot and footwear at 1000 N*s^−1^ (the maximum speed of the MTM) until a maximum vertical force of 2400 N was reached for four consecutive compression loading and unloading cycles. We repeated the same vertical stiffness testing protocol on the prosthetic foot without footwear.

We averaged vertical displacement values across the final three loading cycles for each footwear condition fitted with the prosthetic foot on top of the track surface (∆y_system_) and the prosthetic foot only condition on top of the track surface (∆y_foot_) from 0 to 2400 N. We derived the average vertical displacement of the footwear midsole (∆y_footwear_) based on ∆y_system_ and ∆y_foot_ (Eq. 1).

1 $${\Delta y}_{footwear} = {\Delta y}_{system}- {\Delta y}_{foot}$$

**Fig. 2:**
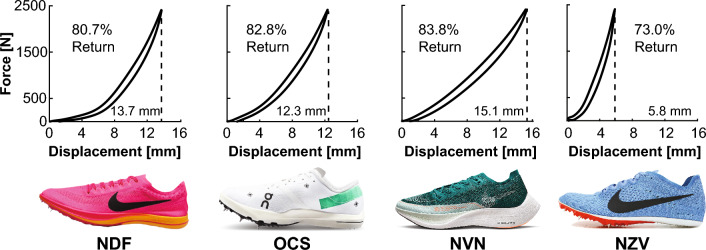
Force–Displacement curves and percentage energy return for the Nike ZoomX Dragonfly AFT spike (NDF), On Cloudspike 10,000 m AFT spike (OCS), Nike ZoomX VaporFly Next % 2 AFT shoe (NVN), and Nike Zoom Victory 3 traditional spike (NZV)

**Fig. 3:**
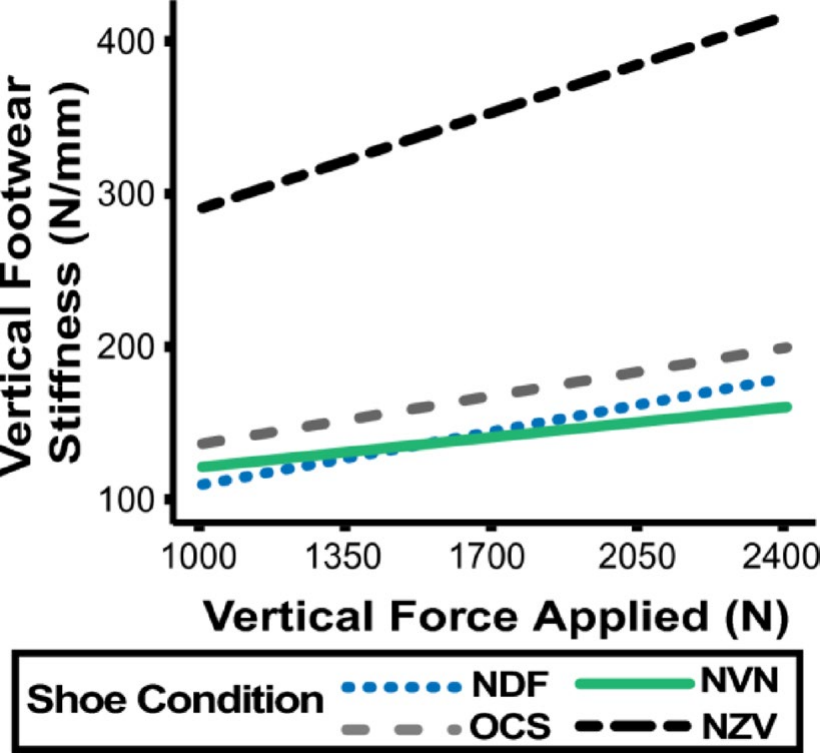
The relationship between vertical force applied and calculated vertical footwear stiffness from the materials testing machine for the Nike ZoomX Dragonfly (NDF) (blue; * k*_*footwear*_ = 0.050 ∗* vGRF*_*peak*_ + 57.4), On Cloudspike 10,000 m (OCS) (grey; * k*_*footwear*_  = 0.045 ∗* vGRF*_*peak*_ + 89.2), Nike ZoomX VaporFly Next % 2 (NVN) (green; * k*_*footwear*_  = 0.028 ∗* vGRF*_*peak*_ + 91.0), and Nike Zoom Victory 3 (NZV) (black; * k*_*footwear*_  = 0.090 ∗* vGRF*_*peak*_ + 198.4)

From the measured force–displacement curves (Fig. 2), we calculated vertical footwear stiffness (k_footwear_) from the quotient of the vGRF and ∆y_footwear_ for each footwear condition from 0 to 2400 N (Eq. 2). Then, we used linear fit models to determine k_footwear_ for each footwear condition for vGRF values ranging from 1300 to 2400 N (Fig. 3).

2 $${k}_{footwear} = \frac{vGRF}{{\Delta \mathrm{y}}_{footwear}}$$

#### Energy return

We calculated the energy stored and returned by the footwear conditions during each loading and unloading cycle from the area under the entire force–displacement curve during compression and extension, respectively (Fig. 2). Then, we calculated the percentage energy return as the energy returned divided by the energy stored for each footwear condition (Fig. 2, Table 2) (Hoogkamer et al. 2018; Alda-Blanco et al. 2024).

**Table 2:** Energy return and vertical stiffness characteristics of each footwear condition

Footwear condition	Energy stored [J]	Energy returned [J]	Energy return [%]	Vertical stiffness [N*mm^−1^]
Nike ZoomX Dragonfly (NDF)	9.7	7.8	80.7	175.3
On Cloudspike 10,000 m (OCS)	10.6	8.8	82.8	195.6
Nike VaporFly Next % 2 (NVN)	14.7	12.3	83.8	158.8
Nike Zoom Victory 3 (NZV)	5.2	3.8	73.0	412.5

Each metric was averaged from three sequential loading–unloading cycles using a materials testing machine from 0 to 2400 N. The vertical stiffnesses reported for each footwear condition were calculated at 2400 N of force application

### Experimental protocol

To measure the physiological and biomechanical effects of competitive runners wearing AFT spikes, AFT shoes, and traditional spikes, we utilized a repeated measures study design. Each participant completed three separate sessions that included a graded exercise test (GXT) and two footwear testing sessions at an elevation of 1655 m (5,430 ft) with at least 48 h between each session. We instructed participants to abstain from ingesting caffeine and alcohol for 12 h and be at least 2 h postprandial prior to the start of each session to mitigate potential effects of diet on metabolic measurements. Additionally, each session was performed at the same time each day to minimize day-to-day variability in metabolic rates. During the GXT, we measured sub-maximal oxygen consumption [ml O_2_*kg^−1^*min^−1^] and the maximum rate of oxygen consumption ($\dot{V}$O_2max_) [ml O_2_*kg^−1^*min^−1^] to ensure that each participant could utilize primarily aerobic metabolism for the duration of each footwear testing trial. For the footwear testing sessions, participants ran wearing four different shoe conditions while we measured their metabolic rates and ground reaction forces and we verified that they had a respiratory exchange ratio < 1.00 (Yeh et al. 1983).

#### Session 1

For the first session, we conducted a running GXT on a level, force-measuring treadmill (Treadmetrix, Park City, UT) to assess sub-maximal oxygen consumption and $\dot{V}$O_2max_ while the participants wore NVN AFT shoes. The results of the GXT were used to verify that each participant could run using primarily aerobic metabolism (RER < 1) during the footwear testing sessions (Yeh et al. 1983). Throughout the test, we measured rates of oxygen uptake and carbon dioxide production using an expired-gas analysis system (TrueOne 2400, Parvo Medics, UT) at standard temperature and pressure, dry (STPD). The sub-maximal portion of the test began at 2.5 m*s^−1^ and, for each subsequent four minute stage, speed was increased by 0.5 m*s^−1^until each participant reached a rating of perceived exertion greater than 16 (Daniels 2013). Participants were then given ten minutes of active or passive rest before beginning the maximal portion of the test. To assess $\dot{V}$O_2max_, each participant completed a maximal effort running test that began at 0.5 m*s^−1^ slower than their final sub-maximal running speed and increased speed by 0.25 m*s^−1^ every minute until volitional exhaustion (Daniels 2013). $\dot{V}$O_2max_ was defined as the highest average rate of oxygen consumption over 30 s during the maximal portion of the test.

#### Sessions 2 and 3

For sessions two and three, participants began with a warm-up of 10 to 20 min of running at a self-selected speed in their personal footwear. Then, each participant completed eight, five-minute running trials on a force measuring treadmill equipped with a 10 mm thick rubber belt that could tolerate spiked footwear while breathing through a mouthpiece connected to an expired-gas analysis system. We measured rates of oxygen consumption and carbon dioxide production at STPD every five seconds throughout each five-minute trial and ground reaction forces (1000 Hz) for thirty seconds during minutes three and four of each five-minute trial. Females ran at 15 km*hr^−1^ and males ran at 17 km*hr^−1^ while using four different pairs of footwear. Since spikes are used for races contested from 18 to 27 km*hr^−1^, we selected the testing speeds because they represented the fastest running speed that our study population could maintain while utilizing aerobic metabolism (RER < 1) (Yeh et al. 1983). The trial order was randomized and mirrored each day so that each participant ran twice wearing each footwear condition during each session (Barrons et al. 2024). Thus, each participant ran wearing each footwear condition for four trials over two days. Between trials, we gave participants five minutes to change footwear and hydrate as necessary, and we measured their body mass while wearing each footwear condition.

### Data analyses

#### Physiological variables

To compare running economy across footwear conditions, we averaged rates of oxygen consumption and carbon dioxide production over the last 2 min of each footwear trial. We used the Péronnet equation (Kipp et al. 2018a; Péronnet and Massicotte 1991) to calculate running economy, defined as the average body mass normalized gross metabolic power while running [W*kg^−1^]. We normalized running economy by each participant’s body mass while wearing each footwear condition measured prior to each trial. Then, we derived the percentage difference in running economy for each footwear condition relative to the average running economy from the NZV traditional spike for each participant. Finally, we averaged the percentage differences in running economy from all participants accounting for footwear condition.

#### Biomechanical variables

We used a 20 Hz 4th order Butterworth low-pass digital filter on the raw ground reaction force data and defined ground contact based on a 20 N vertical ground reaction force threshold. We calculated ground contact time as the time between foot-strike and toe-off, stride length as the horizontal distance traveled from foot-strike to foot-strike of the same foot, and stride frequency as the number of strides taken per second.

Since a runner’s leg can be mechanically modeled using a spring mass system (Farley et al. 1993) and a footwear midsole acts as a spring in series with the runner’s leg, we calculated the combined leg and footwear stiffness (k_system_) from the quotient of the vGRF_peak_ produced during stance and the maximum compression of the leg spring and footwear midsole (∆Y) during stance (Eq. 3).

3 $${k}_{system} =\frac{{vGRF}_{peak}}{\Delta \mathrm{Y}}$$

To determine ∆Y, we measured the participant’s initial leg length, the average distance between the greater trochanter and the ground for each leg while barefoot (L_0_), and the initial footwear stack height at the midfoot (H_0_). We then used L_0_ and H_0_ to calculate theta (θ), which is half of the angle swept by the stance leg, from running velocity (u), ground contact time (t_c_), L_0_, and H_0_ (Eq. 4) (Farley et al. 1993).

4 $$\theta = {sin}^{-1}\left(\frac{u*{t}_{c}}{2*{(L}_{0}+{H}_{0})}\right)$$

Finally, we calculated ∆Y (Eq. 5), which uses the peak vertical displacement of the center of mass (CoM) due to the leg and footwear during ground contact (∆y_system_). ∆y_system_ was calculated by twice integrating the vertical acceleration of the CoM with respect to time during stance (Cavagna 1975).

5 $$\Delta \mathrm{Y}= \Delta {y}_{system}+ {(L}_{0}+{H}_{0})*(1-\mathrm{c}\mathrm{o}\mathrm{s} \uptheta )$$

We mechanically modeled the runner’s leg and footwear midsole as two in-series springs with stiffnesses of k_leg_ and k_footwear_, respectively. We calculated k_footwear_ for each participant by inputting the vGRF_peak_ during each running trial into a linear stiffness model for each footwear condition. We then used the relationship between k_system_, k_leg_, and k_footwear_ (Eq. 6) to solve for k_leg_. After calculating k_leg_, we derived the maximum compression of the leg spring (∆L) as the quotient of vGRF_peak_ and k_leg_ (Eq. 7).

6 $$\frac{1}{{k}_{system}} = \frac{1}{{k}_{leg}}+\frac{1}{{k}_{footwear}}$$

7 $$\Delta \mathrm{L}= \frac{{vGRF}_{peak}}{{k}_{leg}}$$

### Statistics

We used the “lme” function in RStudio to create linear mixed models comparing running economy and biomechanical variables between the four shoe conditions. For all statistical analyses, we used a significance level of p < 0.05. All statistical analyses and data plotting were performed using MATLAB (Natick, MA, USA) and RStudio. Unless otherwise specified, data were reported as mean ± standard deviation.

## Results

**Fig. 4:**
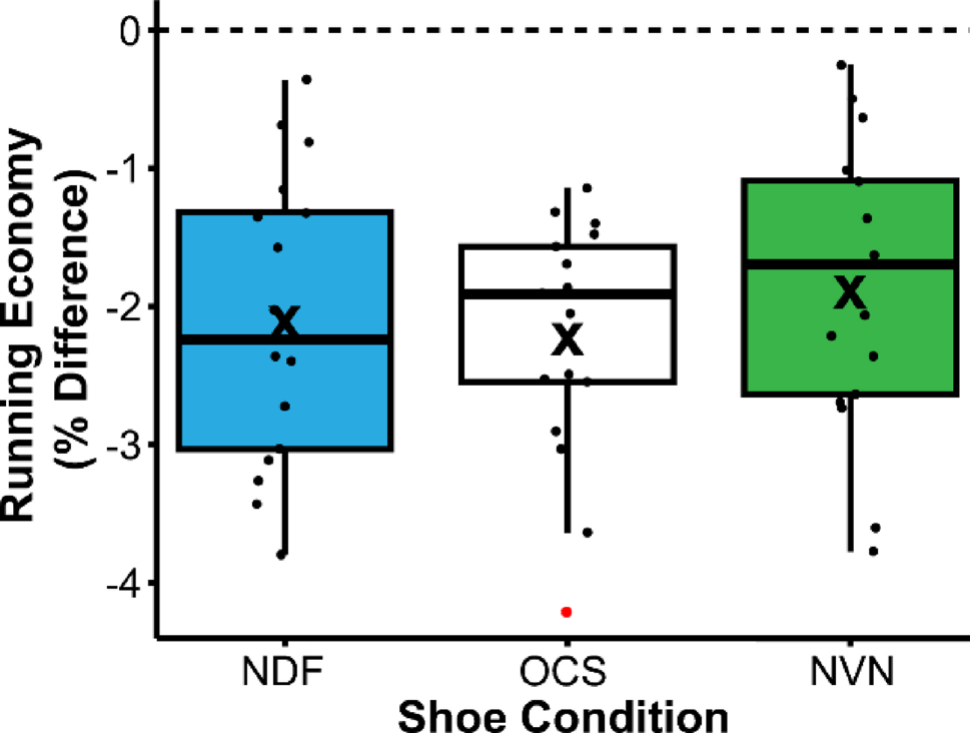
Box and whisker plots of the median (solid black line), mean (black X), interquartile range (box), values within 1.5 times the interquartile range (whiskers), average individual values (black dots), and average individual values outside 1.5 times the interquartile range (red dots) for the percentage difference in running economy [W*kg^−1^] from 17 participants who ran wearing Nike ZoomX Dragonfly (NDF) (blue) and On Cloudspike 10,000 m (OCS) (white) AFT spikes, and Nike ZoomX VaporFly Next % 2 (NVN) (green) AFT shoes compared to Nike Zoom Victory 3 (NZV) traditional spikes. For all AFT shoe conditions, running economy significantly improved compared to NZV traditional spikes (p < 0.001). The dashed horizontal line indicates no percentage change from NZV traditional spikes

Overall, we found that running economy improved (i.e. the metabolic power required to run decreased) by 2.1, 2.3, and 1.9% while runners wore NDF and OCS AFT spikes and NVN AFT shoes compared to NZV traditional spikes (p < 0.001; Fig. 4; Table 3). The average improvement in running economy across all AFT footwear conditions was 2.1% and we found no significant differences in running economy between the NDF, OCS, and NVN AFT footwear conditions. Additionally, we found that the rate of oxygen consumption (V̇O_2_), energetic cost of transport (ECOT), and oxygen cost of transport (O_2_COT) all improved significantly improved while wearing the AFT spike and shoe conditions relative to the NZV traditional spikes (p < 0.001; Table 3).

**Table 3:** Running economy, rate of oxygen consumption (V̇O_2_), energetic cost of transport (ECOT), and oxygen cost of transport (O_2_COT) for 17 participants using different racing footwear

	NDF	OCS	NVN	NZV
Running economy [W*kg^−1^]	18.25 ± 1.84^D^	18.22 ± 1.83^D^	18.29 ± 1.79^D^	18.64 ± 1.86^ABC^
V̇O_2_ [ml O_2_*kg^−1^*min^−1^]	51.65 ± 5.10^D^	51.59 ± 5.06^D^	51.75 ± 4.95^D^	52.66 ± 5.13^ABC^
ECOT [J*kg^−1^*m^−1^]	4.06 ± 0.29^D^	4.05 ± 0.30^D^	4.07 ± 0.28^D^	4.15 ± 0.29^ABC^
O_2_COT [ml O2*kg^−1^*km^−1^]	191.47 ± 13.69^D^	191.30 ± 13.90^D^	191.86 ± 12.98^D^	195.24 ± 13.59^ABC^

Values are reported as mean ± SD. A, B, C, and D superscripts denote significant differences from Nike ZoomX Dragonfly (NDF) AFT spikes, On Cloudspike 10,000 m (OCS) AFT spikes, Nike ZoomX VaporFly Next % 2 (NVN) AFT shoes, and Nike Zoom Victory 3 (NZV) traditional spikes, respectively (p < 0.05)

**Fig. 5:**
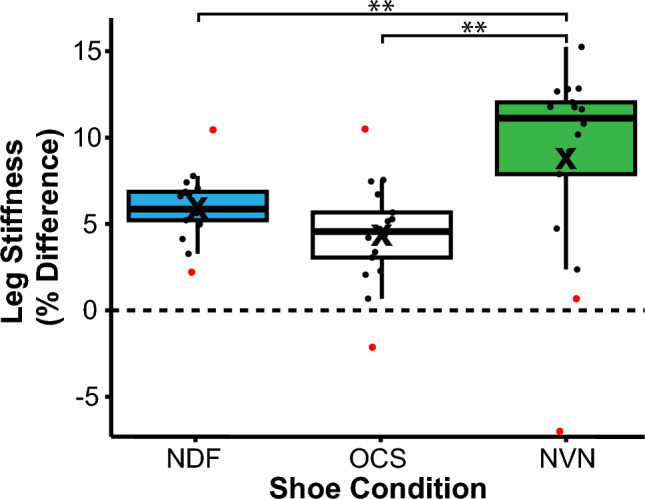
Box and whisker plots of the median (solid black line), mean (black X), interquartile range (box), values within 1.5 times the interquartile range (whiskers), average individual values (black dots), and average individual values outside 1.5 times the interquartile range (red dots) for the percentage difference in leg stiffness [N*mm^−1^] from 17 participants who ran wearing Nike ZoomX Dragonfly (NDF) (blue) and On Cloudspike 10,000 m (OCS) (white) AFT spikes, and Nike ZoomX VaporFly Next % 2 (NVN) (green) AFT shoes compared to Nike Zoom Victory 3 (NZV) traditional spikes. For all AFT conditions, leg stiffness was significantly greater compared to NZV traditional spikes (p < 0.001). The dashed horizontal line indicates no percentage change from NZV traditional spikes. ** denotes a significant difference between AFT conditions (p < 0.05)

We found that leg stiffness increased by 6.1, 4.5, and 8.9% while runners wore NDF and OCS AFT spikes and NVN AFT shoes compared to NZV traditional spikes (p < 0.001; Fig. 5). Leg stiffness was on average 3.7% higher while wearing the NVN AFT shoes compared to NDF and OCS AFT spikes (p < 0.01; Table 4). We found no significant differences in system stiffness (combined leg stiffness and footwear stiffness) and vGRF_peak_ between footwear conditions. However, leg sweep angle, Θ, was an average of 1.3% smaller in the NVN AFT shoes compared to NDF and OCS AFT spikes and NZV traditional spikes (p ≤ 0.004; Table 4). Additionally, ∆L was 2% less while runners wore NDF AFT spikes and NVN AFT shoes compared to NZV traditional spikes and ∆L was 2% less while runners wore NVN AFT shoes compared to OCS AFT spikes (p ≤ 0.017; Table 4).

**Table 4:** Biomechanical variables for 17 participants using different racing footwear conditions

	NDF	OCS	NVN	NZV
Leg stiffness [N*mm^−1^]	19.8 ± 2.7^CD^	19.5 ± 2.8^CD^	20.4 ± 3.1^ABD^	18.6 ± 2.3^ABC^
System stiffness [N*mm^−1^]	17.4 ± 2.1	17.3 ± 2.2	17.7 ± 2.4	17.5 ± 2.1
vGRF_peak_ [BW]	2.89 ± 0.25	2.88 ± 0.25	2.87 ± 0.25	2.88 ± 0.23
Θ [◦]	27.2 ± 1.6^C^	27.3 ± 1.7^C^	26.9 ± 1.9^ABD^	27.1 ± 1.6^C^
∆L [mm]	146 ± 16^D^	149 ± 17^C^	146 ± 19^BD^	149 ± 16^AC^
Ground contact time [ms]	192 ± 12^CD^	194 ± 12^D^	194 ± 13^AD^	189 ± 11^ABC^
Stride length [m]	2.98 ± 0.29^CD^	2.99 ± 0.29^CD^	3.00 ± 0.29^ABD^	2.96 ± 0.28^ABC^
Stride frequency [Hz]	1.51 ± 0.08^CD^	1.51 ± 0.08^CD^	1.50 ± 0.08^ABD^	1.52 ± 0.08^ABC^

Values are reported as mean ± SD. A, B, C, and D superscripts denote significant differences from Nike ZoomX Dragonfly (NDF) AFT spikes, On Cloudspike 10,000 m (OCS) AFT spikes, Nike ZoomX VaporFly Next % 2 (NVN) AFT shoes, and Nike Zoom Victory 3 (NZV) traditional spikes, respectively (p < 0.05)

**Fig. 6:**
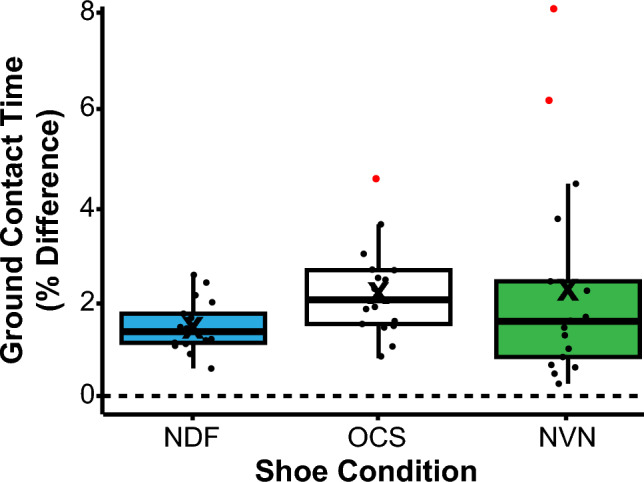
Box and whisker plots of the median (solid black line), mean (black X), interquartile range (box), values within 1.5 times the interquartile range (whiskers), average individual values (black dots), and average individual values outside 1.5 times the interquartile range (red dots) for the percentage difference in ground contact time [ms] for 17 participants who ran wearing Nike ZoomX Dragonfly (NDF) (blue) and On Cloudspike 10,000 m (OCS) (white) AFT spikes, and Nike ZoomX VaporFly Next % 2 (NVN) (green) AFT shoes compared to Nike Zoom Victory 3 (NZV) traditional spikes. For all AFT shoe conditions, ground contact time was significantly greater compared to the NZV traditional spike (p < 0.001). The dashed horizontal line indicates no percentage change from the NZV traditional spike

We found that ground contact time increased by 1.5, 2.2, and 2.3% while runners wore NDF and OCS AFT spikes and NVN AFT shoes compared to NZV traditional spikes (p < 0.001; Fig. 6). Ground contact time decreased by 1% while wearing NDF AFT spikes relative to NVN AFT shoes (p < 0.001; Table 4). We also found that stride length increased and stride frequency decreased by 0.8, 1.1, and 1.5% while runners wore NDF and OCS AFT spikes and NVN AFT shoes compared to NZV traditional spikes, respectively. Additionally, stride length increased and stride frequency decreased by 0.7 and 0.4% while wearing NVN AFT shoes compared to NDF and OCS AFT spikes, respectively (p < 0.001; Table 4).

## Discussion

In the present study, we investigated the running economy and biomechanics of runners wearing AFT spikes and shoes compared to traditional spikes. In support of our first hypothesis, we found that participants wearing the NDF and OCS AFT spikes, and NVN AFT shoes improved their running economy by an average of 2.1% compared to the NZV traditional spikes. Our results are similar to previous studies that found running economy improved by an average of 2 to 4% for runners wearing AFT spikes and shoes compared to traditional racing shoes (Hoogkamer et al. 2018; Hunter et al. 2019; Barnes and Kilding 2019; Joubert et al. 2024). According to a race performance prediction tool developed by Kipp et al., the 2.1% improvement in running economy found in the present study is predicted to improve 10,000 m race performance by 1.4% (Kipp et al. 2019). Although many factors can affect race performance, a 1.4% predicted race performance improvement is in line with the observed 1.3% improvement in the average top 100 male and female 10,000 m race performances since the introduction of AFT spikes (Needles and Grabowski 2024). With these results in mind, athletes wearing AFT spikes and shoes experience significantly improved running economy compared to traditional spikes, and this improvement in running economy could lead to faster race times.

The running economy improvements experienced by runners wearing AFT spikes and shoes may be explained by biomechanical changes. We hypothesized that leg stiffness would increase while runners wore AFT spikes and shoes compared to traditional spikes, and we accept this hypothesis. In agreement with previous studies of running and hopping on compliant surfaces, we found that the combined system stiffness of the runner’s leg and footwear during each trial did not change across footwear conditions (Table 4) (Kerdok et al. 2002; Ferris et al. 1998). However, after accounting for changes in footwear stiffness, leg stiffness increased by an average of 6.5% while wearing NDF and OCS AFT spikes, and NVN AFT shoes (average vertical footwear stiffness: 176.6 N*mm^−1^) compared to NZV traditional spikes (vertical footwear stiffness: 412.5 N*mm^−1^). These results are in line with Kerdok et al. who found that, as treadmill surface stiffness decreased from 454.2 N*mm^−1^ to 216.8 N*mm^−1^, leg stiffness increased by 6.9% while running at 13.3 km*hr^−1^ (Kerdok et al. 2002). We elected to measure leg stiffness since it has been hypothesized that increased leg stiffness at a given running speed leads to a greater effective mechanical advantage (EMA) due to a more upright limb posture (Biewener et al. 2004). An increased EMA necessitates lower joint moments and leg muscle force production, which reduces the activation of the leg muscles and, in turn, improves running economy (Biewener et al. 2004; Kipp et al. 2018b). While we did not estimate EMA or active muscle volume in the present study, Kipp et al. reported that estimated active muscle volume decreased as estimated hip and ankle EMA increased, which presumably coincides with increased leg stiffness (Kipp et al. 2018b). Although active muscle volume does not directly correspond to the amount of metabolic energy a muscle is consuming (Fuglevand et al. 1993), the present results suggest that increased leg stiffness may lead to reduced active muscle volume and, thus, improved running economy while runners wear AFT spikes and shoes compared to traditional spikes.

In the present study, we found significant increases in leg stiffness when competitive runners wore AFT spikes compared to traditional spikes but previous studies have reported potentially contradictory results about the relationship between leg stiffness and AFT footwear. Hata et al. reported no significant change in leg stiffness for participants running at 20 km*hr^−1^ while wearing AFT shoes compared to stiffer traditional shoes (Hata et al. 2022). Moreover, Bertschy et al. found that leg stiffness decreased as footwear compliance increased while running at 14 km*hr^−1^ for runners wearing AFT shoes compared to shoes with stiffer EVA midsoles (Bertschy et al. 2025). These conflicting results may be due to differences in the calculation of leg stiffness. Hata et al. measured change in leg length (∆L) by tracking reflective markers on the center of the hip and ankle (Hata et al. 2022), while in the present study, we used the spring mass model and double integration of the center of mass acceleration to calculate change in leg length (Blickhan 1989; Cavagna 1975; McMahon and Cheng 1990). Bertschy et al. measured change in leg length (∆L) as the change in distance between the greater trochanter and the center of pressure during stance and reported leg stiffness as the combined in-series stiffness of the runner’s leg and footwear midsole (Bertschy et al. 2025). Although Hata et al., Bertschy et al., and the present study each report a value for leg stiffness, the leg stiffness values have been calculated using three distinct methods. Previous research has shown that the relationship between leg stiffness and running speed depends on the method used to calculate leg stiffness (Kuzmeski et al. 2021). So, future research into how the method of calculating leg stiffness affects the relationship between leg stiffness and compliant footwear would allow for a more informed comparison of leg stiffness values across studies. Moreover, future studies that determine the relationship between leg stiffness and running economy are needed to inform footwear design.

In support of our third hypothesis, ground contact time increased by an average of 2.1% while runners wore NDF and OCS AFT spikes and NVN AFT shoes compared to NZV traditional spikes. Our results are in agreement with an analysis of prototype NVN AFT shoes, where Hoogkamer et al. found 0.6% longer ground contact times for athletes wearing AFT shoes compared to traditional marathon footwear while running at 14, 16, and 18 km*hr^−1^ (Hoogkamer et al. 2018). Additionally, Barnes and Kilding reported that ground contact times were 2.5 and 1.6% longer at 14 and 16 km*hr^−1^, respectively, for runners wearing NVN AFT shoes compared to traditional spikes (Barnes and Kilding 2019). Increased ground contact time while wearing AFT spikes and shoes may be due to the inclusion of midsole embedded rigid moderators in AFT footwear. Previous studies found that the addition of a rigid plate above the midsole of running footwear increased ground contact time by 1 to 3% but did not improve running economy compared to the same footwear without a rigid plate (Beck et al. 2020; Flores et al. 2019; Day and Hahn 2020). Longer ground contact times while running could slow the rate of force production by leg muscles, allowing for the recruitment of more economical muscle fibers that require less metabolic energy (Kram and Taylor 1990). However, since the addition of rigid plates above the midsole of running footwear resulted in increased ground contact time without changes in running economy, further research is necessary to investigate if increased ground contact time due to the midsole embedded rigid plates in AFT spikes and shoes has a meaningful impact on running economy and performance.

When combined, increased leg stiffness and ground contact time may provide a biomechanical explanation for improved running economy while wearing AFT spikes and shoes compared to traditional spikes. Kipp et al. reported that 98% of the increase in metabolic rate as running speed increased from 8 to 18 km*hr^−1^ could be explained by increased active leg muscle volume and shorter ground contact time (Kipp et al. 2018b). Additionally, in the same study, hip and ankle EMA decreased as active muscle volume increased across the range of speeds tested (Kipp et al. 2018b). Thus, based on the assumption that increased leg stiffness leads to increased EMA and reduced active muscle volume, the combined changes in leg stiffness and ground contact time may be key contributors to improved running economy while wearing AFT spikes and shoes. However, Kram and Taylor, Kipp et al., and the present study analyzed the stance phase mechanics of running to predict changes in running economy and did not assess changes in running economy based on the swing phase of running (Kipp et al. 2018b; Kram and Taylor 1990). Previous studies have estimated that the swing phase of running accounts for ~ 20% of total net metabolic rate at 3 m/s (Modica and Kram 2005). Accordingly, future research that utilizes work-based models that account for the stance and swing phases of running to estimate running economy may reveal further potential biomechanical mechanisms that lead to improved running economy while wearing AFT spikes and shoes.

Due to the potential running economy and performance benefits of compliant, high energy return AFT footwear (Hoogkamer et al. 2018; Healey et al. 2022; Burns and Joubert 2024), we characterized the mechanical energy return of each footwear condition used in our study. We found that NDF and OCS AFT spikes and NVN AFT shoes returned 7.8, 8.9, and 12.4 J of mechanical energy per step, respectively, compared to just 3.8 J from the NZV traditional spikes (Fig. 2, Table 2). The two to three times increase in mechanical energy return for AFT spikes and shoes relative to traditional spikes is likely due to the compliant and resilient PEBA midsole foams and rigid plates used in AFT spikes and shoes. The midsole embedded rigid plates used in AFT footwear have been shown to store and return a negligible amount of mechanical energy when bent or compressed while running (Hoogkamer et al. 2019). However, the interaction of a midsole embedded rigid plate and midsole foam results in a 1.3 to 5.2% increase in footwear mechanical energy return compared to the same midsole without a rigid plate (Perry et al. 2025; Ghanbari et al. 2025). Thus, the combination of PEBA midsole foams and midsole embedded rigid plates likely leads to an increase in mechanical energy return while wearing AFT spikes and shoes compared to traditional spikes with EVA midsole foams. While running, leg muscles require metabolic energy to generate forces that facilitate elastic energy storage and return in tendons and muscles (Smith et al. 2005). It has been hypothesized that mechanical energy return from footwear and surfaces may reduce the required force production and energy storage of muscles and tendons while running (Hoogkamer et al. 2018; Ferris et al. 1998; Frederick et al. 1986). As a result, the increased mechanical energy storage and return capabilities of AFT footwear may lead to improved running economy and race performance for athletes wearing AFT spikes and shoes.

In 2024, World Athletics, the international governing body of track and field, banned the use of footwear with midsole stack heights exceeding 20 mm for track races (World Athletics 2021). Despite this new regulation categorizing NVN AFT shoes (40 mm stack height) as illegal for World Athletics sanctioned track races, a comparison of the NDF and OCS AFT spikes and NVN AFT shoes may provide further insights for the development of AFT spikes. NVN AFT shoes return an average of 4 J more mechanical energy per step, are ~ 15% more compliant, and resulted in ~ 3.7% higher leg stiffness values compared to the AFT spike conditions (Tables 2 & 4). Based on these findings, we expected that runners wearing NVN AFT shoes would elicit improved running economy relative to the AFT spike conditions, but we found no significant difference in running economy between the AFT spikes and AFT shoes (Table 3). This contradiction may be explained by the differences in footwear mass between the AFT spikes and shoes. Previous studies have found that increasing footwear mass by 100 g worsens running economy by 0.8 to 1.2% (Hoogkamer et al. 2016; Franz et al. 2012; Frederick et al. 1984) and, on average, the NVN AFT shoes were 90 g heavier than the AFT spikes. If we had weight-matched the AFT spike conditions to the mass of the NVN AFT shoes, we would expect that the NVN AFT shoes would improve running economy by ~ 0.9% relative to the AFT spike conditions. Therefore, while our results suggest that increased footwear energy return and compliance may improve running economy, increasing midsole energy return and compliance at the expense of increased footwear mass may reduce the performance benefits of AFT spikes.

There are some potential limitations that may have affected the interpretation of the results for the present study. Contrary to some previous AFT shoe studies (Hoogkamer et al. 2018; Barnes and Kilding 2019), we elected to not weight match the footwear conditions in the present study. The NVN AFT shoes were an average of 140 g heavier than the NZV traditional spikes and running economy worsens by ~ 1% for every 100 g of mass added to a shoe (Hoogkamer et al. 2016; Franz et al. 2012; Frederick et al. 1984). However, competition spikes and shoes are not required to be weight-matched during races and, to allow generalizability, we did not modify the mass of the AFT spikes and shoes used in the present study. Moreover, the footwear conditions utilized in the present study are designed for races that are typically run at average speeds of 18 to 27 km*hr^−1^ at the elite level, while we collected data at 15 and 17 km*hr^−1^ to ensure athletes were primarily utilizing aerobic metabolism. Previous AFT footwear research has been conducted at running speeds from 14 to 18 km*hr^−1^ and there has been no reported effect of running speed on running economy improvements experienced by athletes wearing AFT shoes relative to traditional shoes within this speed range, which allows comparisons between studies (Hoogkamer et al. 2018; Barnes and Kilding 2019; Joubert et al. 2024). However, there may be an effect of running speed on the race performance of runners wearing AFT spikes from 18 to 27 km*hr^−1^ (Needles and Grabowski 2024). Accordingly, future studies of AFT spikes conducted at faster running speeds may be needed to determine the effects of AFT spikes and running speed on race performance.

## Conclusion

Overall, we found that runners wearing AFT spikes and shoes improved their running economy by an average of 2.1% and increased their leg stiffness and ground contact time by an average of 6.5% and 2.1%, respectively, compared to traditional spikes. With our findings in mind, the next generation of AFT spikes could be designed with increased midsole compliance and energy return to further improve running economy and performance by eliciting potentially favorable biomechanical outcomes such as increased leg stiffness and ground contact time.

## Data Availability

All data collected for the study are available upon request from the corresponding author.

## Abbreviations

AFT: Advanced footwear technology
NDF: Nike ZoomX Dragonfly
OCS: On Cloudspike 10,000 m
NVN: Nike Vaporfly Next % 2
NZV: Nike Zoom Victory 3
MTM: Materials testing machine
GXT: Graded exercise test
STPD: Standard temperature and pressure, dry
EMA: Effective mechanical advantage

## Code availability

The code used to process the data collected during this study is available upon request from the corresponding author.
